# Evaluation of peripheral blood eosinophilia, serum ECP levels of eosinophils in inflammatory infiltrates of gastric mucosa in food allergy patients

**DOI:** 10.1186/2045-7022-1-S1-P83

**Published:** 2011-08-12

**Authors:** Malgorzata Graczyk, Zbigniew Bartuzi, Michal Przybyszewski, Katarzyna Napiórkowska, Magdalena Zbikowska-Gotz, Jacek Tlappa, Ewa Szynkiewicz, Robert Zacniewski, Ewa Socha

**Affiliations:** 1 Collegium Medicum in Bydgoszcz, Department of Allergology, Clinical Immunology and Internal Diseases, Bydgoszcz, Poland

## Introduction

Eosinophilic infiltration may be found in all segments of the gastrointestinal tract. The goal of this study was to evaluate the peripheral blood eosinophilia, serum ECP levels and presence of eosinophils in inflammatory infiltrates of the gastric mucosa in food allergy patients.

## Materials and methods

80 patients were enrolled in the study, including 50 patients with food allergy and 30 patients with dyspeptic discomfort without underlying food allergy. All subjects were subjected to gastroscopic examination, gastric mucosa biopsy and Helicobacter pylori colonization status. Blood was collected from each subject for determination of peripheral blood eosinophilia and ECP levels.

## Results

The presence of eosinophils in gastric mucosa from food allergy patients was found in 42% of subjects, and an eosinophil count of >10 per field was observed in as many as 20% of patients. Helicobacter pylori colonization was observed in 38% of subjects suffering from food allergy and in 60% of patients with dyspeptic symptoms without concomitant allergy. Arithmetic average serum ECP levels in food allergy patients was 24.604 +/- 40.36 μg/L. The average serum ECP levels in allergy-free patients was 29.9 +/- 64.76 μg/L. The average peripheral blood eosinophil count determined in the study population of food allergy patients was 221.34 +/- 175 eos/mm2. In patients with dyspeptic symptoms without concomitant food allergy, the average peripheral blood eosinophilia was 121.4 +/- 100.75 eos/mm2.

## Conclusions

The study showed that the presence of eosinophils in gastric mucosa was more prevalent in food allergy patients compared to patients without food allergies; however, the difference was not statistically significant. Analysis of the results revealed no statistically significant differences between serum ECP levels in food allergy patients and patients with dyspeptic symptoms without concomitant food allergy. Both study groups showed a statistically significant differentiation of average peripheral blood eosinophil counts. The statistically significant positive correlation was found between the ECP levels and peripheral blood eosinophilia in food allergy patients.

